# Cardiac Myxoma- A case series from a Cardiothoracic Unit in a developing country: Sri Lanka

**DOI:** 10.1186/1749-8090-10-S1-A285

**Published:** 2015-12-16

**Authors:** Muditha N Lansakara, Mohomad N Jazeel, Athula Wijethunga, Kumaradasan Gnanakanthan, Samath D Dharmaratne

**Affiliations:** 1 Department of Cardiothoracic Surgery, Teaching Hospital, Kandy, Sri Lanka; 2 Department of Community Medicine, Faculty of Medicine, University of Peradeniya, Sri Lanka and Institute for Health Metrics and Evaluation, Department of Global Health, School of Public Health, University of Washington, Seattle, WA, USA

## Background/Introduction

Myxomas remain the most common benign primary cardiac tumors, where it's growth can masquerade as mitral stenosis, infective endocarditis and collagen vascular disease. This tumour is rare with an estimated incidence of 0.0067% - 0.33%. They can lead to embolisation, conduction disturbances and lethal valve obstructions.

## Aims/Objectives

To describe socio-demographic characteristics, presentation and outcome of cardiac myxoma patients presenting to our Cardiothoracic Unit.

## Method

All patients who presented and underwent surgical excision at our Cardiothoracic Unit, and histologically proven as cardiac myxoma were included over a period of six years from January 2009. Data were collected from the operation note data base and the patient records and was analyzed by SPSS statistical software.

## Results

There were 31 patients who underwent surgery. Their age ranged from 15 years to 69 years with a mean age of 43.2 years. The majority (71%) were females. The main presenting symptoms were shortness of breath (43.5%) and stroke (34.8%). No one died from the surgery and no one suffered significant morbidity. All patients had myxomas in the left atrium where majority were attached to the inter atrial septum (80.5 %) Almost all recovered from strokes without residual effects

## Discussion/Conclusion

By early diagnosis and prompt surgery, myxoma can be treated with minimal complications with the desired outcome. Sometimes a high degree of suspicion is required for early diagnosis.

